# Can muscle avert GLP1R weight plateau and regain?

**DOI:** 10.1016/j.xcrm.2025.102308

**Published:** 2025-09-16

**Authors:** Dongdong Wang, Andre Djalalvandi, Christina T. Saed, Katherine M. Morrison, Gregory R. Steinberg

**Affiliations:** 1 Centre for Metabolism, Obesity and Diabetes Research, McMaster University, Hamilton, ON L8N 3Z5, Canada; 2 Division of Endocrinology and Metabolism, Department of Medicine, McMaster University, Hamilton, ON L8N 3Z5, Canada; 3 Department of Paediatrics, McMaster University, Hamilton, ON L8S 4L8, Canada

## Abstract

GLP1R-based obesity therapies can reduce lean muscle and energy expenditure via adaptive thermogenesis (also known as metabolic adaptation), leading to weight plateaus and regain. Defining the role of muscle energy expenditure in mediating these effects is critical to improving next-generation treatments and sustaining long-term weight loss.

## Main text

Obesity results from an imbalance of energy intake, absorption, and expenditure of as little as 10–30 kcal per day.^1^ It significantly increases the risk of metabolic, cardiovascular, and oncological diseases. Obesity is also associated with higher rates of depression and anxiety although the molecular underpinnings of this relationship remain less well understood. Despite the multifaceted etiology for these comorbidities, longitudinal studies studying reduced caloric intake and exercise have established long-term health benefits of weight loss. However, a persistent challenge with the management of obesity through diet and exercise is sustainability, as most individuals will regain weight within 1–2 years and less than 6% of adults with lifestyle-induced weight loss will maintain it over 15 years.^2^ Therefore, understanding the mechanisms driving weight loss plateaus and subsequent weight regain is important not only for sustaining weight loss but also preventing its related comorbidities resulting from weight regain.

GLP1R agonists, such as semaglutide, lower body weight primarily by reducing energy intake^3^ without affecting energy expenditure (EE). This suppression of energy intake and subsequent reduction in adiposity significantly lowers liver steatosis and decreases the incidence of T2D and kidney disease.^2^ An emerging concern is the identified reduction in lean muscle mass that has been observed, similar to other interventions that target reduced caloric intake. Furthermore, emerging real-world evidence indicates that discontinuation of semaglutide after 12 months is relatively common (∼50%),^4^ and not surprisingly often leads to rapid weight regain within a year. Similar findings regarding weight loss plateaus or weight regain have been observed with the GLP1-GIP receptor dual agonist Tirzepatide (The SURMOUNT-4 Randomized Clinical Trial, NCT04660643), GLP1-glucagon receptor dual agonist Mazdutide (First Phase 3 Clinical Trial of Mazdutide, NCT05607680), and GLP1-GIP-glucagon tri-agonist Retatrutide (Phase 2 Trial of Retatrutide for Obesity, NCT04881760). A recent study (NCT04081337) demonstrated that the GLP-1/GIP receptor dual agonist Tirzepatide reduced both sleeping energy expenditure (EE) and total energy expenditure (TEE) following weight loss, even after adjusting for fat-free mass and fat mass. This suggests that either GLP-1 receptor agonists or GLP-1/GIP receptor dual agonists do not prevent adaptive thermogenesis following weight loss.

In individuals with obesity, weight regain or relapse is influenced by three variables: energy intake, energy absorption, and energy expenditure. The first two variables, energy intake and absorption, are driven by a complex interplay of physiological, psychological, and environmental factors that have been discussed in depth, recently.^5^ In addition to altering energy intake and absorption, weight loss also directly influences total energy expenditure (TEE). It has been recognized for 30 years that weight loss induced by dietary restriction reduces TEE, findings which have been confirmed in multiple studies using a variety of distinct methodologies (whole room indirect calorimeter, ventilated hood indirect calorimeter, doubly labeled water). This reduction in TEE is also observed following bariatric surgery. However, an ongoing debate remains as to whether this reduction in TEE is greater than or simply proportional to the loss of fat mass (FM) and fat-free mass (FFM). When the reduction in TEE and its components (resting and non-resting EE) exceeds what would be expected based on body composition changes, this phenomenon is referred to as adaptive thermogenesis (AT) or metabolic adaptation.^6^ Importantly, irrespective of the intervention or methodology used to assess TEE, longitudinal studies in individuals with obesity following weight loss clearly indicate that weight loss leads to TEE reductions and this generally ranges from ∼10% to 20% of total energy expenditure before weight loss.^7^ Understanding the cause of this reduction in TEE is critical for developing more sustainable weight loss solutions.

**Figure 1:**
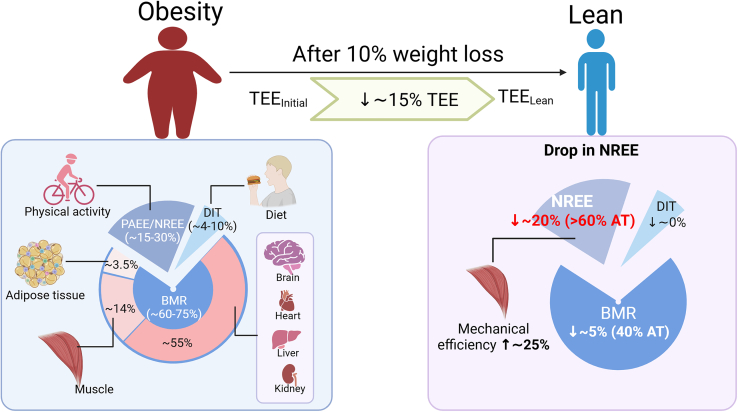
The adaptive thermogenesis during weight loss Total energy expenditure (TEE) decreases by approximately 15% following a 10% weight loss, even after adjusting for body weight or fat-free mass. Reductions in body mass or fat-free mass account for about 60% of the decline in energy expenditure (EE), while the remaining ∼40% is attributed to adaptive thermogenesis (AT). Of this, basal metabolic rate (BMR) decreases by around 5%, contributing roughly 40% to AT, whereas non-resting energy expenditure (NREE) declines by approximately 20%, accounting for the remaining 60% of AT. This may be attributed to an approximately 25% improvement in the mechanical efficiency of skeletal muscle. DIT, diet-induced thermogenesis; PAEE, physical activity energy expenditure. (Figure created using Biorender, agreement number XV28IL3BIG.)

TEE is influenced by both energy intake and absorption and is a function of three primary components: basal metabolic rate (BMR), diet-induced thermogenesis (DIT), and physical activity energy expenditure (PAEE) or non-resting energy expenditure (NREE = TEE − (BMR+DIT)). In sedentary individuals, BMR constitutes about 60%–75% of daily EE and is primarily driven by vital functions of the brain, heart, liver, and kidneys despite these organs comprising only 5%–6% of total body weight (Figure 1). The remaining ∼20% of BMR is derived from skeletal muscle metabolism and ∼5% from adipose tissue. In most studies, reductions in body mass explains approximately 60% of the decline in EE,^8^ largely due to losses in skeletal muscle mass and organ mass. DIT accounts for approximately 10% of TEE and varies with the macronutrient content of the diet, reflecting the energy consumed by digestion and nutrient absorption. However, after adjusting for fat-free mass, very few studies have observed significant changes in BMR or DIT after weight loss.^9^^,^^10^ These data suggest that reductions in these variables are unlikely to be the primary drivers of adaptive thermogenesis.

The most variable component of TEE is PAEE or NREE which includes structured exercise and spontaneous physical activities like walking and fidgeting.^11^ In most studies that have reported significant adaptive thermogenesis, NREE accounts for most of the variance from predicted EE changes following weight loss.^12^ Specifically, during a 10% weight loss, TEE decreases by 8.2–11.1 kcal/day/kg FFM, with NREE reductions accounting for 62.2%–71.9% of this drop (5.9–6.9 kcal/day/kg FFM), highlighting NREE as a major driver of TEE reduction. This reduction in NREE has been observed in studies with doubly labeled water and indirect calorimetry,^13^ with the latter revealing residual values of NREE—calculated as the difference between observed energy expenditure and predicted values derived from regression equations incorporating FFM, FM, and age prior to weight loss—that persist in patients with sustained weight loss.^13^ This supports the concept that a reduction in NREE is likely independent of body mass loss, but it is important to note that not all studies have confirmed this finding. In addition to caloric restriction, GLP1-based therapeutics also decrease NREE and PAEE during weight loss, by ∼170 kcal/day, even when the weight loss is only 2 kg. These data suggest that the weight loss plateau and rapid weight regain after discontinuation of treatment may be caused by reductions in NREE.

So, what is the mechanism for reduced NREE? The primary tissue contributing to a PAEE/NREE is skeletal muscle^8^ and improved skeletal muscle work efficiency may be important. Theoretically, increased skeletal muscle work efficiency would equate to reduced oxygen consumption for a given workload during exercise, leading to lower EE. Specifically, a 10% reduction in body weight has been found to improve gross mechanical efficiency of skeletal muscle during non-weight bearing cycling exercise by ∼25%, independent of changes in skeletal muscle mass.^14^ A 10% decline in weight would lead to an ∼20% decrease in NREE,^14^ which could equate to ∼200 kcal/day (assuming 1,000 kcal NREE/day). Importantly, this reduction in NREE therefore accounts for 50%–70% of the total decrease in TEE (300–400 kcal/day) following weight loss. Mechanistically, we have recently shown in mice that this improvement in efficiency may be related to reduced calcium futile cycling.^15^ Thus, the decline in skeletal muscle energy expenditure during physical activity, potentially driven by increased muscle work efficiency, may be the primary contributor to adaptive thermogenesis following weight loss.

Therefore, while GLP1R-based medications such as Semaglutide and Tirzepatide have revolutionized obesity treatment, there is still a significant unmet need to maximize long-term and sustainable weight loss. Addressing this challenge will likely require strategies that preserve or enhance energy expenditure pathways to prevent weight loss plateau and weight regain. Future studies examining the mechanisms contributing to reduced PAEE and improvements in muscle efficiency may open the door to new classes of therapeutics that assist with sustainable interventions for obesity and metabolic disease.

## Acknowledgments

G.R.S. acknowledges the support of a Diabetes Canada Investigator Award (OG-3-22-5645-GS), Canadian Institutes of Health Research Foundation Grant (201709FDN-CEBA-116200), a Tier 1 Canada Research Chair in Metabolic Diseases, and a J. Bruce Duncan Endowed Chair in Metabolic Diseases.

## Declaration of interests

G.R.S. is a co-founder and shareholder of Espervita Therapeutics. McMaster University has received funding from Cambrian Biosciences, Catalym, Espervita Therapeutics, Esperion Therapeutics, Poxel Pharmaceuticals, Merck, Nestle, and Novo Nordisk for research conducted in the laboratory of G.R.S. G.R.S. has received consulting/speaking fees from Curie Bio, Eli Lilly, Korro Bio, Keros Therapeutics, Merck, Novo Nordisk, Poxel Pharmaceuticals, and Versant Ventures. K.M.M. is an advisory board member for Novo Nordisk and Medison Therapeutics and is a member of a data safety monitoring board for Novartis. The other authors declare no competing interests.
